# Movement of the RecG Motor Domain upon DNA Binding Is Required for Efficient Fork Reversal

**DOI:** 10.3390/ijms19103049

**Published:** 2018-10-06

**Authors:** Garrett M. Warren, Richard A. Stein, Hassane S. Mchaourab, Brandt F. Eichman

**Affiliations:** 1 Department of Biological Sciences, Vanderbilt University, Nashville, TN 37232, USA; garrett.m.warren@vanderbilt.edu; 2 Department of Molecular Physiology and Biophysics, Vanderbilt University, Nashville, TN 37232, USA; richard.a.stein@vanderbilt.edu (R.A.S.); hassane.mchaourab@vanderbilt.edu (H.S.M.)

**Keywords:** DNA replication, DNA repair, DNA damage response, DNA translocation, DNA helicase, superfamily 2 ATPase, replication restart, fork reversal, fork regression, chromatin remodeler

## Abstract

RecG catalyzes reversal of stalled replication forks in response to replication stress in bacteria. The protein contains a fork recognition (“wedge”) domain that binds branched DNA and a superfamily II (SF2) ATPase motor that drives translocation on double-stranded (ds)DNA. The mechanism by which the wedge and motor domains collaborate to catalyze fork reversal in RecG and analogous eukaryotic fork remodelers is unknown. Here, we used electron paramagnetic resonance (EPR) spectroscopy to probe conformational changes between the wedge and ATPase domains in response to fork DNA binding by *Thermotoga maritima* RecG. Upon binding DNA, the ATPase-C lobe moves away from both the wedge and ATPase-N domains. This conformational change is consistent with a model of RecG fully engaged with a DNA fork substrate constructed from a crystal structure of RecG bound to a DNA junction together with recent cryo-electron microscopy (EM) structures of chromatin remodelers in complex with dsDNA. We show by mutational analysis that a conserved loop within the translocation in RecG (TRG) motif that was unstructured in the RecG crystal structure is essential for fork reversal and DNA-dependent conformational changes. Together, this work helps provide a more coherent model of fork binding and remodeling by RecG and related eukaryotic enzymes.

## 1. Introduction

**Figure 1:**
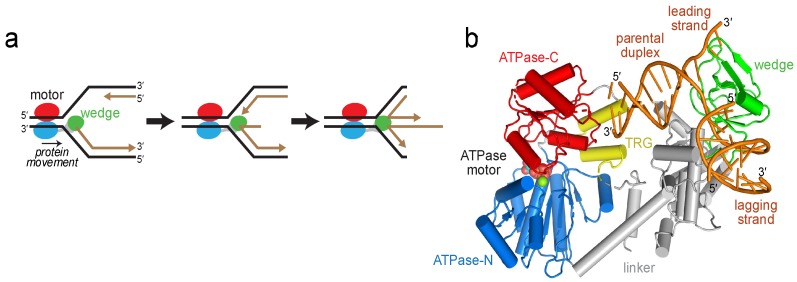
RecG catalyzes replication fork reversal. (**a**) Schematic of fork reversal. Template DNA strands are black and nascent strands are brown. RecG is colored according to domains: ATPase-N and -C lobes are blue and red, respectively, and the wedge domain is green. (**b**) Crystal structure of RecG bound to fork DNA, Protein Data Bank (PDB) ID 1GM5. The protein is colored as in panel a, with the translocation in RecG (TRG) motif yellow and DNA orange.

Faithful DNA replication at every round of cell division is critical for transmission of genetic information. Replisomes assembled at progressing replication forks regularly encounter a number of impediments including DNA damage, aberrant DNA structures, difficult to replicate nucleotide sequences, and transcription complexes [1]. Stalled replication forks can lead to replisome disassembly, strand breaks and other pathogenic DNA structures, and are a potential source of genome instability associated with a number of diseases [1,2]. To ensure complete genome duplication, a number of pathways operate to mitigate fork stalling or to restart replication through reassembly of the replication fork in an origin independent manner [3,4]. One important mechanism for stabilizing or restarting stalled forks is fork reversal (or fork regression), in which specialized motor proteins push the fork backward to convert the three-way fork into a four-way junction (Figure 1a) [5,6,7,8]. The Holliday junction-like structure serves as an important intermediate for recombination-coupled repair and can also promote template switching to enable DNA synthesis from an unhindered nascent strand template [3]. Fork reversal may also promote excision repair of fork-stalling DNA lesions by sequestering them away from the fork and back into the context of dsDNA.

Fork reversal mechanisms are operative in both prokaryotes and eukaryotes [3,7,8]. In bacteria, the dsDNA translocase RecG is a key player in this process and is important for maintenance of genome stability via DNA repair and recombination [9,10,11]. Inactivation of RecG sensitizes cells to the interstrand crosslinking agent mitomycin C and to UV and ionizing radiation [12,13], and leads to over-replication of the terminus region in circular DNA [14,15]. The molecular rationale for these phenotypes remains under debate [16], but may result from the generation of DNA structures necessary for origin-independent replication restart by PriA [9,10,17,18] or recombination repair by RecA/RecBCD or RuvABC machinery [9,19,20].

In vitro, RecG catalyzes regression of replication forks and branch migration of Holliday junctions [21,22], even in the presence of stalled replisome components [23], and also unwinds D-loops and R-loops [24,25,26]. These remodeling activities rely on ATP-dependent dsDNA translocation catalyzed by a superfamily 2 (SF2) helicase motor comprised of two RecA-like ATPase lobes [27]. RecG preferentially binds Holliday junctions and model replication forks that contain ssDNA on the leading strand and dsDNA on the lagging strand [28,29]. The basis for RecG’s preference for branched structures was illustrated by a crystal structure of the *Thermotoga maritima* enzyme bound to a model replication fork, which revealed an N-terminal oligonucleotide/oligosaccharide (OB)-fold (“wedge”) domain that engaged both leading and lagging template strands at the branch point, and that is connected to the motor by a helical linker (Figure 1b) [30]. DNA remodeling is presumably catalyzed by dsDNA translocation by the motor tracking with 3′→5′ polarity on the lagging strand of the parental duplex toward the fork [29,31], while the wedge domain aids unwinding of parental-nascent duplexes and possibly annealing of nascent strands to form the four-way Holliday junction [30,32] (Figure 1a).

How the motor domain engages DNA and how translocation is coupled to fork stabilization by the wedge domain to remodel a branched nucleic acid substrate is not entirely clear, in part because the DNA corresponding to the parental duplex template in the structure was too short to contact the ATPase motor (Figure 1b). One clue for DNA translocation was provided by the identification of a conserved helical hairpin—the TRG (translocation in RecG) motif—in RecG and TRCF/Mfd (transcription-repair coupling factor), a bacterial SF2 helicase that translocates on dsDNA to terminate transcription [33,34,35,36]. Mutagenesis of the TRG motif impaired fork reversal by RecG and displacement of RNA polymerase from DNA by TRCF/Mfd, and thus this motif is essential for DNA translocase activities in both proteins [33,34]. In RecG, the TRG motif is centrally located between the wedge and motor domains, but the TRG region predicted to lie in the path of the DNA was disordered in the crystal structure, and thus how it enables DNA translocation remains speculative [33,35,37,38].

In this study, we aimed to understand the role of the TRG motif and how the RecG motor engages parental DNA in the context of a fork. Using a combination of electron paramagnetic resonance (EPR) spectroscopy and mutagenesis, we found that *T. maritima* RecG undergoes a conformational change in the ATPase motor relative to the wedge domain upon binding a model DNA replication fork. DNA binding is required to activate the ATPase activity and fork reversal activity, and therefore our EPR distance distributions provide insight into the operation of a DNA fork remodeling enzyme fully bound to a relevant DNA substrate in solution. In addition, we expanded on the previous TRG analysis [33] by showing that the conserved loop region C-terminal to the TRG motif is critical for ATP hydrolysis and fork reversal activity, and that mutations in the loop attenuate conformational changes induced by DNA binding. Our data support a model whereby the TRG loop is required for stabilizing the DNA-bound motor in an active conformation.

## 2. Results

### 2.1. Reorientation of the RecG Motor Domain to Accommodate the Parental DNA Duplex

**Figure 2:**
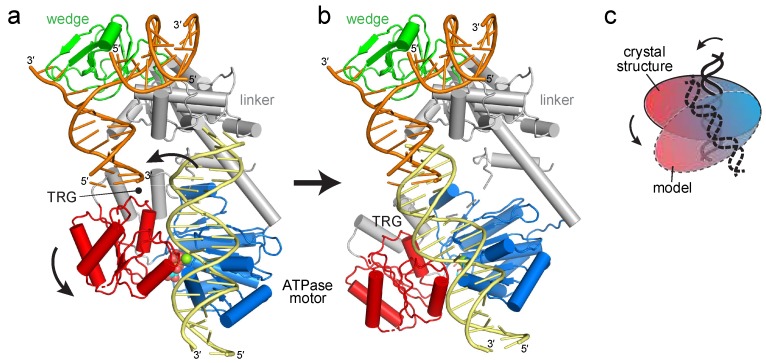
Reorientation of the RecG motor domain to accommodate parental DNA. (**a**) The RecG/DNA crystal structure (PDB ID 1GM5), rotated 90° with respect to the view shown in Figure 1b. The wedge domain is colored green, the linker domain is grey, and the ATPase motor is blue (N-lobe) and red (C-lobe). Parental DNA (yellow) was modeled by superposition of the XPB-ATPase and its bound DNA from the TFIIH complex (PDB ID 5IY9) onto the RecG-ATPase domain. The curved black arrow denotes the rotation of the motor domain necessary to align the helical axis of the modeled DNA to that of the crystal structure. (**b**) Model of RecG bound to parental DNA after 30° rotation of the RecG motor and its accompanying DNA. (**c**) Schematic of the rotation of the motor domain needed to bring parental duplex into alignment with the fork.

The RecG crystal structure illustrated how the wedge domain engages the branch point of a DNA fork [30], but did not address the interaction of the motor domain with DNA or its relative conformation in the DNA-bound state because the 10 base pairs (bps) of parental duplex used in the structure did not reach the motor domain (Figure 1b). The structure predicts that at least 25 bps are necessary to fully engage the motor, consistent with DNase I footprinting showing that RecG protects a significant portion of the parental DNA duplex [39]. To gain insight into how the motor and wedge domains might collaborate in a fully bound DNA complex, we constructed a model of DNA bound to the motor domain using available structures of SF2 ATPase motors bound to dsDNA (Figure 2a, Supplemental Figure S1). Recent cryo-EM structures of chromatin remodeling complexes CHD1, SNF2, INO80 bound to nucleosomes [40,41,42,43,44] and of Xeroderma pigmentosum B (XPB) helicase within the TFIIH component of the transcription pre-initiation complex [43] showed a conserved path of DNA across the N- and C-terminal lobes of the ATPase in a manner predicted from an archaeal Rad54 homolog bound to DNA in an open conformation [45]. Superposition of the DNA from these structures onto RecG using the motor domain as a guide shows that the modeled and crystalized DNA duplexes are misaligned (Figure 2a). Alignment of these two DNA segments into a continuous parental duplex requires either a 25–40° bend in the DNA helical axis or rotation of the motor domain in which the ATPase-C lobe swings away from the wedge domain (Figure 2b,c).

**Figure 3:**
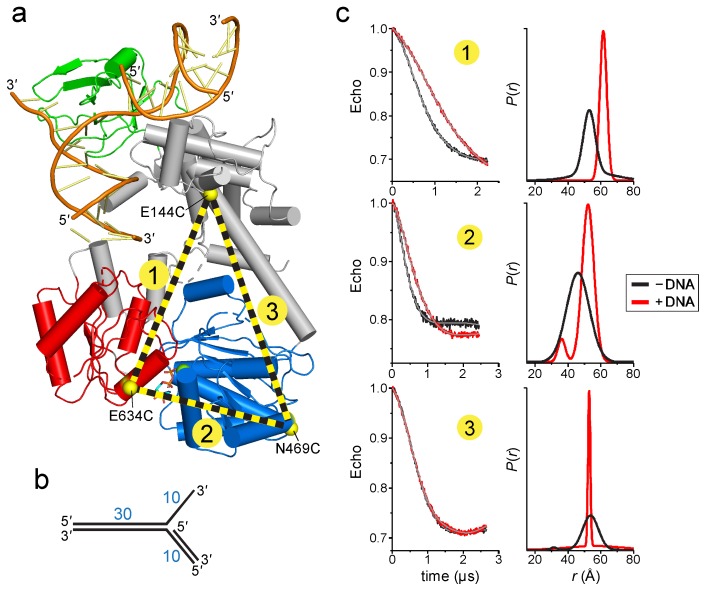
RecG changes conformation upon binding DNA. (**a**) Cα carbons of MTSL-labeled cysteines are shown as yellow spheres and labeled on the RecG/DNA crystal structure (PDB ID 1GM5). MTSL pairs 1 (E144-E634), 2 (N469-E634), and 3 (E144-N469) are shown as yellow-black dashed lines. (**b**) Schematic of the DNA fork used in electron paramagnetic resonance (EPR) experiments. (**c**) Double electron-electron resonance (DEER) data for MTSL pairs 1, 2, and 3 in the absence (black) and presence (red) of DNA. **Left**, pairwise time domain data. **Right**, individual fits of the DEER data shown as a probability distribution (*P*) as a function of interatomic distance (*r*).

To determine if DNA binding causes a conformational change within the protein, we used electron paramagnetic resonance (EPR) to determine the distances between domains upon addition of DNA. The four-pulse, double electron-electron resonance (DEER) technique provides probability distributions of the distances between spin-labeled residue pairs [46]. Our experimental design was to place spin-labels in three domains—the linker that connects the wedge to the ATPase motor, the ATPase N-lobe connected to the linker, and the ATPase C-lobe (Figure 3a). The linker region is predicted to be relatively inflexible based on the network of centrally located α-helices, whereas the C-lobe is likely more mobile given its peripheral location. We used the *Thermotoga* RecG protein for our experiments in order to correspond to the crystal structure [30]. The spin label (1-oxy-2,2,5,5-tetramethyl-pyrolline-3-methyl)-methanethiosulfonate (MTSL) was introduced at positions Glu144, Asn469, and Glu634, which were chosen on the basis of their surface exposed locations. After substitution of native cysteine residues to serine, non-native cysteines were introduced pairwise to produce E144C-E634C (pair 1), N469C-E634C (pair 2), and E144C-N469C (pair 3) mutants necessary for thiol conjugation of MTSL (Figure 3a). We verified that neither the Cys mutations nor the spin-labels affected the DNA dependent ATPase activity of the protein (Figure S2a,b). Continuous wave (CW) spectra of each MTSL-RecG protein were consistent with surface exposed sites (Figure S2c).

DEER data were collected in the absence and presence of a DNA fork similar to that crystalized but containing a 30-nucleotide parental duplex region (Figure 3b), long enough to span the motor domain (Figure 2b). In the absence of DNA, the distance distributions were consistent with those predicted from the crystal structures. The DEER traces for pairs 1 and 2 exhibited a significant change upon addition of DNA that are described by an ~10 Å increase in the center of the distance distribution and a decrease in the disorder as judged by a decrease in the width of the distance distribution (Figure 3c). This shift is consistent with the conformation change shown in Figure 2, whereby the C-lobe moves away or rotates relative to both the N-lobe and the linker. In contrast, the DEER traces for pair 3 were nearly identical in the absence and presence of DNA. The resultant pair 3 distance distributions were not identical but did not indicate any shift in the median distance, suggesting that the N-lobe does not move away upon addition of DNA. Taken together, the DEER measurements provide evidence for a RecG conformational change upon binding to a model replication fork and are consistent with the rotation of the ATPase domain predicted from our model (Figure 2).

### 2.2. Mutation of the TRG Motif Attenuates RecG Conformational Changes upon DNA Binding

**Figure 4:**
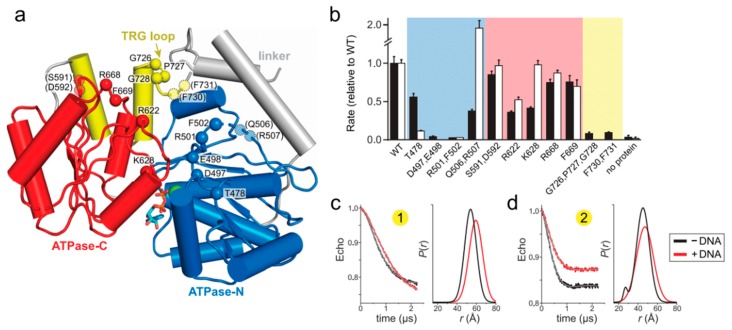
Loops within the TRG motif are essential for DNA-dependent ATP hydrolysis and fork reversal activity. (**a**) Structure of the ATPase domain (blue and red) with residues lining the putative DNA binding surface shown as Cα spheres. The TRG hairpin and loop are colored yellow. Dashed lines represent disordered regions in the crystal structure. (**b**) Relative DNA-dependent ATP hydrolysis (black bars) and fork reversal activities (white bars) of alanine mutants. Shading corresponds to the location of each mutant in the structure shown in panel a. Raw data and rates are shown in Figure S3. (**c**,**d**) DEER measurements for spin-label pairs 1 (**c**) and 2 (**d**) in the TRG loop mutant, G726A P727A G728A. Pairwise time domain data and individual fits of the DEER data are shown on the left and right of each panel, respectively.

To gain additional insight into how RecG’s motor domain engages DNA, we carried out a mutational analysis of residues predicted from our model to bind DNA. The parental DNA duplex is predicted to contact both N- and C-lobes of the ATPase domain and the TRG loop, which is part of the linker connecting the ATPase motor and wedge domains (Figure 2a and Figure 4a). Importantly, the putative DNA binding cleft contains several loops that were disordered in the crystal structure, presumably because of the absence of bound DNA. We thus tested the functional importance of residues within these disordered regions, among others. Residues along the predicted DNA binding cleft, as well as those known to be involved in ATP hydrolysis, were mutated to alanine and the mutant proteins tested for DNA-dependent ATPase and fork reversal activities (Figure 4b and Figure S3). None of the mutants showed a difference in DNA binding affinity relative to wild-type as measured directly using fluorescence polarization or electrophoretic mobility shift assays, consistent with previous mutational analysis of *Escherichia coli* RecG [33], presumably because tight binding of the wedge domain to the DNA junction masked any potential modest disruption in duplex DNA binding by the motor domain mutants [32]. Because previous biochemical characterization of RecG has focused on the *E. coli* enzyme, we verified that the fork reversal activities of the *T. maritima* and *E. coli* enzymes are comparable (Figure S4).

Within the ATPase domain, residues in the N-lobe were found to have the most significant effects on RecG activity. We tested residues within motifs Ic and II, which in SF2 helicases are responsible for DNA binding (motif Ic) and ATP binding and hydrolysis (motif II) [47,48,27]. Alanine substitution of the conserved Thr478 in motif Ic led to a significant (10-fold) decrease in fork reversal activity without significantly affecting ATPase activity (Figure 4b), consistent with results from *Mycobacterium tuberculosis* RecG and RNA helicase NS3 [49,50]. Also consistent with other helicases, mutation of motif II in *T. maritima* RecG (D497A E498A) completely abolished both fork reversal and ATPase activities (Figure 4b). Residues immediately C-terminal to motif II are conserved across RecG proteins and have been suggested to be important allosteric regulators of DNA-dependent ATP hydrolysis in *E. coli* PriA and RecQ [51,52]. Our RecG R501A F502A double mutant abrogated ATPase and fork reversal activities, likely because it disrupted the active site. Alanine substitution of Gln506 and Arg507, which were disordered in the RecG structure, had a much weaker effect on ATPase and fork reversal activities (Figure 4b). Similarly, mutation of residues in the ATPase C-lobe did not have a substantial effect on either ATP hydrolysis or fork reversal. Of the residues we tested, the largest effect was observed from mutation of conserved basic amino acid residues Arg622 and Lys628 within motif IVa (Figure 4b), which participates in nucleic binding in SF2 helicases and is in close proximity to the DNA backbone in the THFIIH, INO80, and SNF2 structures [40,41,42,43].

In contrast to the SF2 motor domain, mutation of the TRG motif had the most severe impact on RecG function. The TRG motif contains a highly conserved loop that was unstructured in the RecG structure and that lies directly in the proposed path of DNA binding [30]. Two separate mutants of this loop (G726A P727A G728A and F730A F731A) abrogated fork reversal and ATP hydrolysis (Figure 4b). Loss of activity by these mutants indicates that the TRG loop is important for binding DNA during translocation, facilitating interdomain movement by the motor during the ATPase cycle, or both. Indeed, the TRG loop lies at the intersection of the two ATPase lobes and the wedge domain, directly in the proposed path of DNA and near helicase motifs III and VI, which coordinate ATP hydrolysis and translocation (motif III) and facilitate ATP binding and hydrolysis (motif VI) in other SF2 helicases [27,48].

To test the role of the TRG loop in RecG DNA-dependent conformation changes, we used EPR to measure interdomain distances in the dysfunctional TRG loop mutant, G726A P727A G728A. Spin labels were introduced into the mutant in the same location as the wild-type protein. We hypothesized that if the TRG loop mediates DNA binding or the DNA-induced conformational change observed in the wild-type protein, then addition of DNA to the mutant would not affect the distance distributions. Indeed, the increase in spin label pair 1 distance upon addition of DNA was reduced without the concomitant decrease in disorder compared to wild-type (Figure 4c and Figure S2d). The TRG loop mutation showed an even greater effect on spin label pair 2, from which only a modest shift in distance was observed upon addition of DNA (Figure 4c and Figure S2d). Therefore, we conclude that the loop C-terminal to the TRG motif mediates DNA-induced conformational changes within the motor, and likely couples motor domain dynamics to the fork-binding wedge domain to drive translocation.

## 3. Discussion

Coupling of an SF2 motor to a fork recognition domain is a conserved feature in the eukaryotic fork remodelers SMARCAL1, HLTF, and ZRANB3 [53,54,55], and thus it is important to understand how the two domains collaborate to drive fork reversal. By extrapolation from ssDNA translocation mechanisms of SF1 and SF2 helicases, the current model for dsDNA translocation by the fork and chromatin remodelers entails conversion of an open to closed conformation of ATPase lobes upon binding DNA [44,45,56]. DNA duplex binding along the interface of the two ATPase lobes places the tracking (3′→ 5′) strand in contact with motif Ia in the ATPase-N lobe and motif IV in the ATPase C-lobe. Consequently, ATP-induced conformational changes between the two ATPase lobes would drive an inchworm movement of the tracking strand and concomitant rotary motion of the duplex [57]. As the fork recognition domain keeps the protein anchored to the junction [32], DNA translocation would effectively pull the unwound template strands back into the protein, facilitating their annealing to each other and unwinding from nascent strands as they encounter the junction. This collaboration between motor and fork binding domains is analogous to INO80 chromatin remodeling machinery, which uses the ARP5 subunit to bind both histone and DNA in order to position the INO80 motor to pump DNA into the nucleosome [41,40]. Both mechanisms require an anchor point to grip the substrate to facilitate productive translocation by the motor.

Our EPR results revealed a DNA-induced movement of RecG’s ATPase-C lobe relative to the positions of the ATPase-N lobe and the wedge domain. This motion can be modeled by a simple pivoting of the motor at the ATPase-N lobe, or a more complex rotation between the two ATPase lobes. The range of motion that we observe between RecG’s two ATPase lobes is not as dramatic as that observed in fluorescence resonance energy transfer studies of an archaeal homolog of Rad54, a related SNF2-like dsDNA translocase [56]. Although we cannot say with certainty the nature of the open and closed conformations of the motor domain from our distance measurements, the two ATPase lobes in the ADP-bound crystal structure are already well-positioned to accommodate dsDNA in a catalytic orientation. The motion of the motor with respect to the wedge that we observe is more striking, since it is clear that the relative positions of the motor and wedge in the crystal structure cannot support a contiguous parental DNA duplex without a rotation of the motor or a sharp bend in the helical axis of the DNA. The latter is unlikely since coupling motor activity to fork stabilization by the wedge domain would place tension on the DNA segment between the two domains. Moreover, the position of the motor domain observed in the crystal structure is constrained by a neighboring protein molecule in the crystal that pushes the motor closer to the wedge. Thus, our data supports a conformational transition from a more compact state in the absence of DNA to a more extended state upon engaging a fork.

Our mutational analysis of the relatively unstructured DNA binding surface of the ATPase domain is consistent with and extends the previous studies showing the TRG motif to be essential for RecG function [33]. The previous mutational analysis focused on the helical hairpin itself, but it is the loop extending from the C-terminal end of the helical hairpin that resides in the path of the DNA and at the intersection of the motor and wedge domains, and that is likely the mechanical element directly responsible for DNA translocation. It was hypothesized that an ATP-induced conformational change in the TRG helical hairpin, propagated through motif VI, would restructure the TRG loop to act as a lever or ratchet to mechanically move or stabilize the DNA in a new conformation [33]. This TRG loop is highly conserved among RecG and Mfd orthologs, with the consensus sequence G(P/A/V)Gd ФФGxxQ(S/T)G (where Ф is a hydrophobic residue). Mutation of the invariant glutamine (Q640) in *E. coli* RecG demonstrated that the TRG loop was essential for RecG activity in vivo [33]. We now show by mutation of the GPG and ФФ residues in the *T. maritima* enzyme that this loop is essential for ATPase and fork reversal activities. More importantly, we found that disruption of the GPG sequence curtailed the range of DNA-induced interdomain motion, implying that this loop region is important for coupling motor and wedge domains. We hypothesize, based on our DEER distance measurements, that the TRG motif loop is required to stabilize an activated conformation of the ATPase domains upon DNA binding to promote ATP hydrolysis [33], similar to the postulated role of the brace helix in the chromatin remodelers [40,41,42,44,58]. In those structures, the brace helix spans the two ATPase lobes and likely stabilizes a closed conformation through interaction of hydrophobic residues on the brace helix and the ATPase N-lobe. It may be that the conserved hydrophobic residues in the TRG loop that are essential for RecG activity may help to organize the two ATPase lobes in a similar manner.

## 4. Materials and Methods

All experiments were carried out using *T. maritima* RecG containing a C-terminal hexahistidine tag (TmRecG-His_6_). We verified that addition of the His_6_ tag did not affect enzyme activity (Figure S4).

### 4.1. Protein Purification

TmRecG-His_6_ was overexpressed from a pET28a^+^-*TmrecG* vector [59] in *E. coli* Tuner (DE3) cells at 37 °C for 3 h in Lysogeny broth (LB) medium supplemented with 100 μg/mL kanamycin and 500 μM isopropyl β-D-1 thiogalactopyranoside (IPTG). Cells were lysed by sonication in buffer containing 50 mM Tris pH 7.5, 600 mM NaCl, 20% glycerol (*v*/*v*), 1 mM dithiothreitol (DTT), 1 mM phenylmethylsulfonyl fluoride, 0.5 µg/ml leupeptin, and 0.5 µg/ml aprotinin. The lysate was clarified by centrifugation at 50,000× *g* at 4 °C for 45 min. RecG-His_6_ was purified by nickel nitrilotriacetic acid (Ni-NTA) agarose affinity chromatography in buffer containing 50 mM Tris pH 7.5, 600 mM NaCl, 25 mM imidazole, 5% glycerol, and 1 mM tris(2-carboxyethyl)phosphine (TCEP) and eluted in buffer containing 50 mM Tris pH 7.5, 600 mM NaCl, 250 mM imidazole, 5% glycerol, 1 mM TCEP. RecG-His_6_-containing fractions were subjected to heparin sepharose chromatography using a 0.1–1 M NaCl gradient in buffer containing 50 mM Tris pH 7.5, 100 mM NaCl, and 15% glycerol.

Mutant RecG expression vectors were generated using the Q5 mutagenesis kit (New England Biolabs) and sequence verified prior to use. All mutant proteins were overexpressed the same as wild-type protein. Alanine mutants were purified by Ni-NTA affinity chromatography, flash frozen, and stored at −80 °C in buffer containing 50 mM Tris pH 7.5, 600 mM NaCl, 250 mM imidazole, 5% glycerol (*v*/*v*), and 1 mM DTT. To prepare cysteine mutants for spin-labeling, all five native cysteines in RecG were first mutated to serine to generate a Cys-less RecG, which was then used to generate three separate double mutants (E144C N469C, E144C E634C, and N469C E634C). Cysteine mutant proteins were purified using Ni-NTA and heparin chromatography and stored at −80 °C in buffer containing 50 mM Tris pH 7.5, 600 mM NaCl, and 10% glycerol (*v*/*v*). Spin-labeling was carried out by incubating cysteine mutants with a 20-fold molar excess of MTSL for 2 h at room temperature, followed by addition of another 20-fold molar excess of MTSL and incubation for 2 h at room temperature and then overnight at 4 °C. Excess MTSL was removed using a HiTrap Sephadex G-25 desalting column (GE Healthcare, Chicago, IL, USA) in buffer containing 50 mM Tris pH 7.5, 500 mM NaCl, and 10% (*v*/*v*) glycerol.

To test the effect of the C-terminal His_6_-tag, we generated a cleavable pET-28a/RecG-3C-His_6_ construct in which the His_6_-tag could be removed with Rhinovirus 3C protease. Q5 mutagenesis kit (New England Biolabs, Ipswich, MA) was used to replace the sequence K^776^LIEVG^781^*KLAAALE* (non-native residues italicized) in the pET28a^+^-*TmrecG* vector with the 3C recognition sequence LEVLFQGP. Proteolytic cleavage generates a 781-residue protein with I^775^*LEVLFQ* sequence at the C-terminus. RecG-3C-His_6_ protein was overexpressed and purified the same as TmRecG-His_6_. The His_6_-tag was removed by a 16-hr incubation with 3C protease after elution from the Ni-NTA column.

*E. coli* RecG was purified from a pGS772-RecG expression plasmid [21] as previously described [60], with an added heparin-sepharose purification step at the end.

### 4.2. EPR

Spin-labeled TmRecG-3C-His_6_ protein was buffer exchanged using Amicon Ultra 15 mL centrifugal units 30 kDa MWCO (MilliporeSigma, Burlington, MA, USA) into buffer containing 50 mM Tris pH 7.5, 100 mM NaCl, and 30% (*w*/*v*) glycerol. Fork DNA was prepared by annealing strands F1/F2/F3 (Table 1) in SSC buffer (15 mM sodium citrate pH 7.0 and 150 mM NaCl). A 2-fold molar excess of DNA was added to 25–50 µM protein and the complex flash frozen in liquid nitrogen. DEER experiments were performed at 83 K on a Bruker 580 pulsed EPR spectrometer at Q-band frequency (33.5 GHz) using a standard four-pulse protocol [61]. Analysis of the DEER data to determine *P*(*r*) distance distributions was carried out using homemade software running in MATLAB [62,63].

### 4.3. ATPase Assay

TmRecG-His_6_ proteins were dialyzed against reaction buffer (50 mM Tris pH 7.5, 50 mM NaCl, and 5 mM MgCl_2_) prior to use. An immobile Holliday junction with 30-bp arms was prepared by annealing the oligodeoxynucleotides J1/J2/J3/J4 (Table 1) in SSC buffer. ATPase reactions (100 μL) were carried out in reaction buffer and contained 50 nM TmRecG-His_6_, 100 nM DNA, 1 mM ATP, 3 mM phosphoenol pyruvate (PEP), 437 μM nicotinamide adenine dinucleotide, 15.75–24.5 U/mL l-lactate dehydrogenase, 10.5–17.5 U/mL pyruvate kinase, and 1 mM DTT. Absorbance at 340 nm was monitored at 25 °C in 96-well plates using a Biotek Synergy H1 hybrid multimode microplate reader. Absorbance was recorded every 60 s for 1 h.

### 4.4. Fork Reversal Activity

Fork reversal activity was measured as previously described [54] with minor modifications. Reactions were performed in reaction buffer and contained 200 pM RecG and 1 nM ^32^P-labeled DNA fork substrate (Table 1). Reactions were quenched at various times (0, 5, 10, 20, 30, 60, and 120 min) by adding proteinase K (Sigma-Aldrich, St. Louis, MO, USA) to a final concentration of 1 mg/mL and incubating for 10 min. Reactions were brought to 5% glycerol (*v*/*v*) and 0.1% bromophenol blue prior to electrophoresis on an 8% non-denaturing polyacrylamide gel at 5 W for 3 h. Gels were exposed overnight to a phosphor plate and bands quantified by autoradiography using a Typhoon Trio and ImageQuant 7.0 software (GE Healthcare, Chicago, IL, USA).

## Figures and Tables

**Table 1:** Oligodeoxynucleotides used in this study. ^1^

**EPR**
F1—(^32^P)GGTCAGTCCTGTCTTCGGCAAAGCTCCATGATCATTGGCA
F2—CGCCGGGCCGCATGGAGCTTTGCCGAAGACAGGACTGACC
F3—CGGCCCGGCG
**ATPase**
J1—GGGTGAACCTGCAGGTGGGCCAGCTCCATGATCATTGGCAATCGTCAAGCTTTATGCCGT
J2—CGATGGACACGTCTTATGTGTGCAGTGCTCGCATGGAGCTGGCCCACCTGCAGGTTCACCC
J3—CATGTAGCGGCTGGCGTCTTAAAGATGTCCCGAGCACTGCACACATAAGACGTGTCCATCG
J4—ACGGCATAAAGCTTGACGATTGCCAATGATGGACATCTTTAAGACGCCAGCCGCTACATG
**Fork Reversal ^2^**
F48—(^32^P)ACGCTGCCGAATTCTACCAGTGCCTTGCTAGGACATCTTTGCCCACCTGCAGGTTCACCC
F50—GGGTGAACCTGCAGGTGGGCAAAGATGTCC
F52—GGGTGAACCTGCAGGTGGGCAAAGATGTCCCAGCAAGGCACTGGTAGAATTCGGCAGCGTC
F53—GGACATCTTTGCCCACCTGCAGGTTCACCC

^1^ Colors denote homologous regions. ^2^ Mismatch (underlined) placed at the junction to prevent spontaneous branch migration.

## Supplementary Materials

Supplementary materials can be found at http://www.mdpi.com/1422-0067/19/10/3049/s1.

## Abbreviations

ATP	adenosine 5′-triphosphate
ATPase	adenosine triphosphatase
DEER	double electron-electron resonance
DTT	dithiothreitol
EDTA	ethylenediaminetetraacetic acid
EPR	electron paramagnetic resonance
MTSL	[1-oxy-2,2,5,5-tetramethyl-pyrolline-3-methyl]-methanethiosulfonate
NTA	nitrilotriacetic acid
SF2	superfamily 2
SRD	substrate recognition domain
SSC	saline-sodium citrate
TCEP	tris(2-carboxyethyl)phosphine
TRG	translocation in RecG
