# Supplementary material for: Movement of the RecG Motor Domain upon DNA Binding Is Required for Efficient Fork Reversal
Source: Int J Mol Sci. 2018 Oct 6;19(10):3049. doi: 10.3390/ijms19103049 (PMC6213257; doi:10.3390/ijms19103049)
Supplement: Supplementary file 1 [file ijms-19-03049-s001.pdf]

Supplemental Information

Movement of the RecG motor domain upon DNA binding is required for efficient fork reversal

Garrett M. Warren, Richard A. Stein, Hassane S. Mchaourab, and Brandt F. Eichman

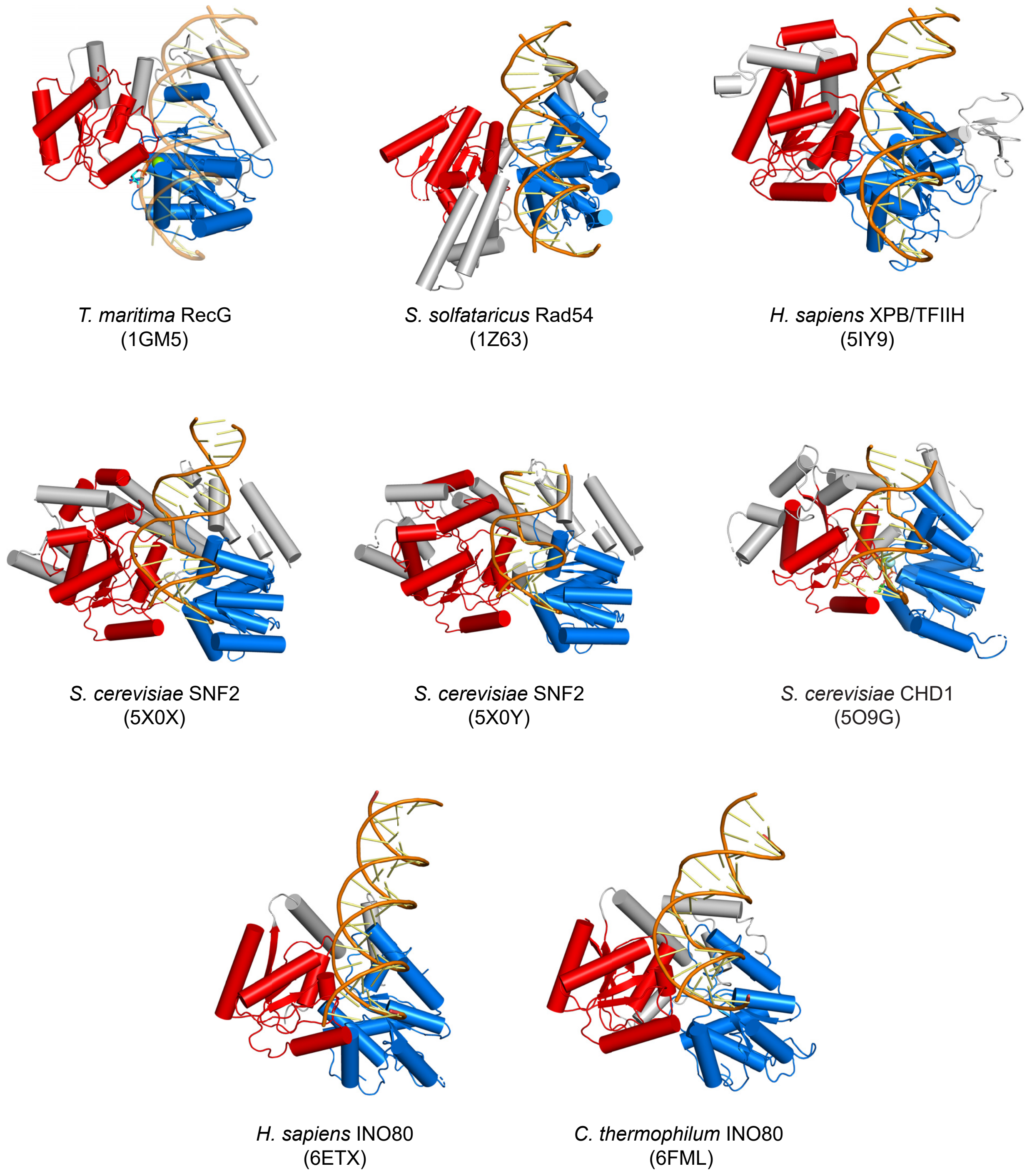

Figure S1. Duplex DNA binding by SF2 family remodelers. ATPase motor domains and the region of bound duplex DNA are shown from each structure. ATPase-N and -C subdomains are colored blue and red, respectively. Structures are aligned by their ATPase-N lobes. PDB ID codes are shown in parentheses below each structure. DNA bound to RecG is was modeled from the XPB/TFIIH structure.

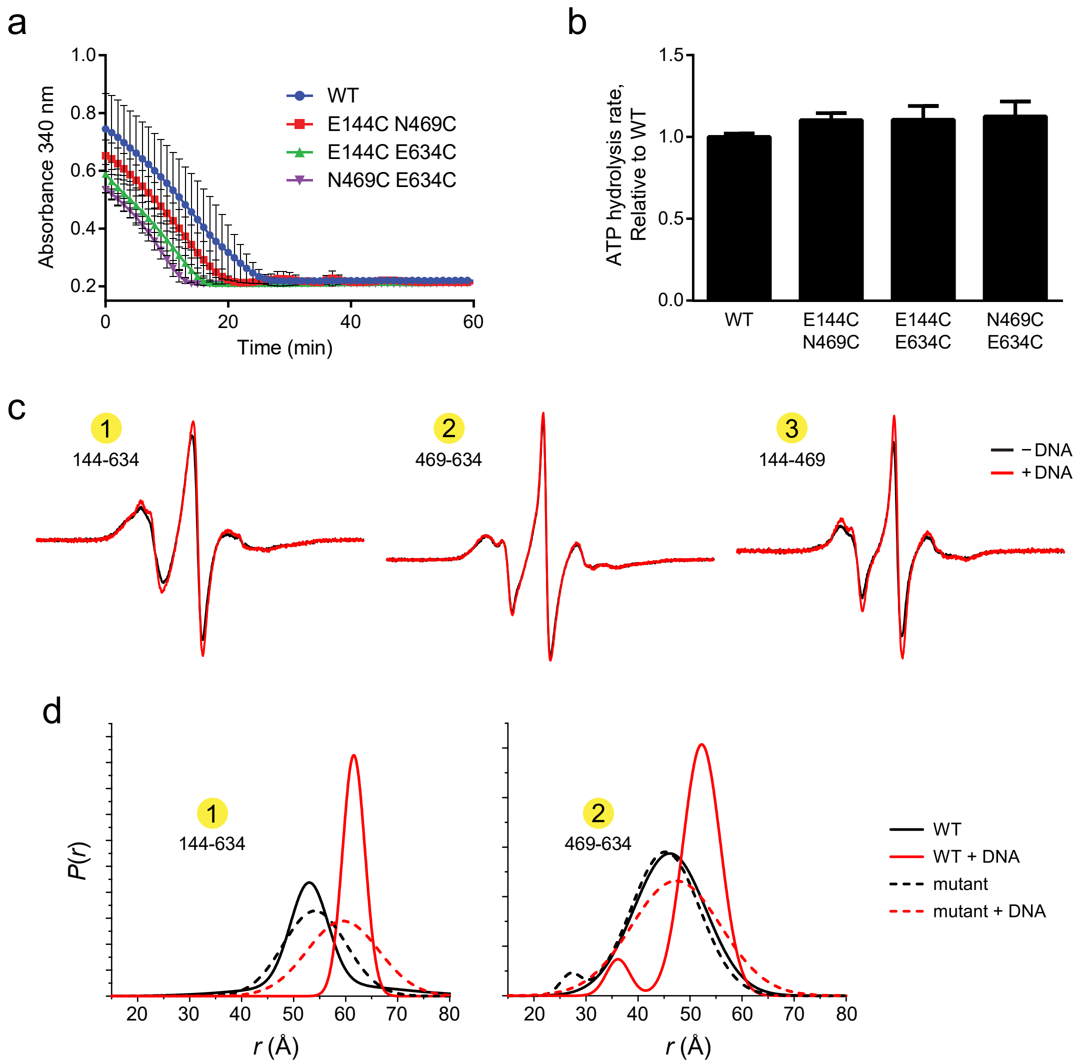

Figure S2. Activity of MTSL-labeled RecG proteins. (a) Raw ATPase data from an NADH coupled assay of spin-label mutants. (b) Relative ATPase activities of spin-label mutants relative to wild-type RecG, determined from the slopes in panel a. (c) Overlayed CW spectra for MTSL pairs 1 (E144-E634), 2 (N469-E634), and 3 (E144-N469) in the absence (black) and presence of DNA (red). (d) Probability distributions for MTSL pairs 1 (E144-E634, *left*) and 2 (N469-E634, *right*) in wild-type (solid lines) and the G726A P727A G728A TRG loop mutant (dashed lines). Distributions for protein alone are black and RecG-DNA are red.

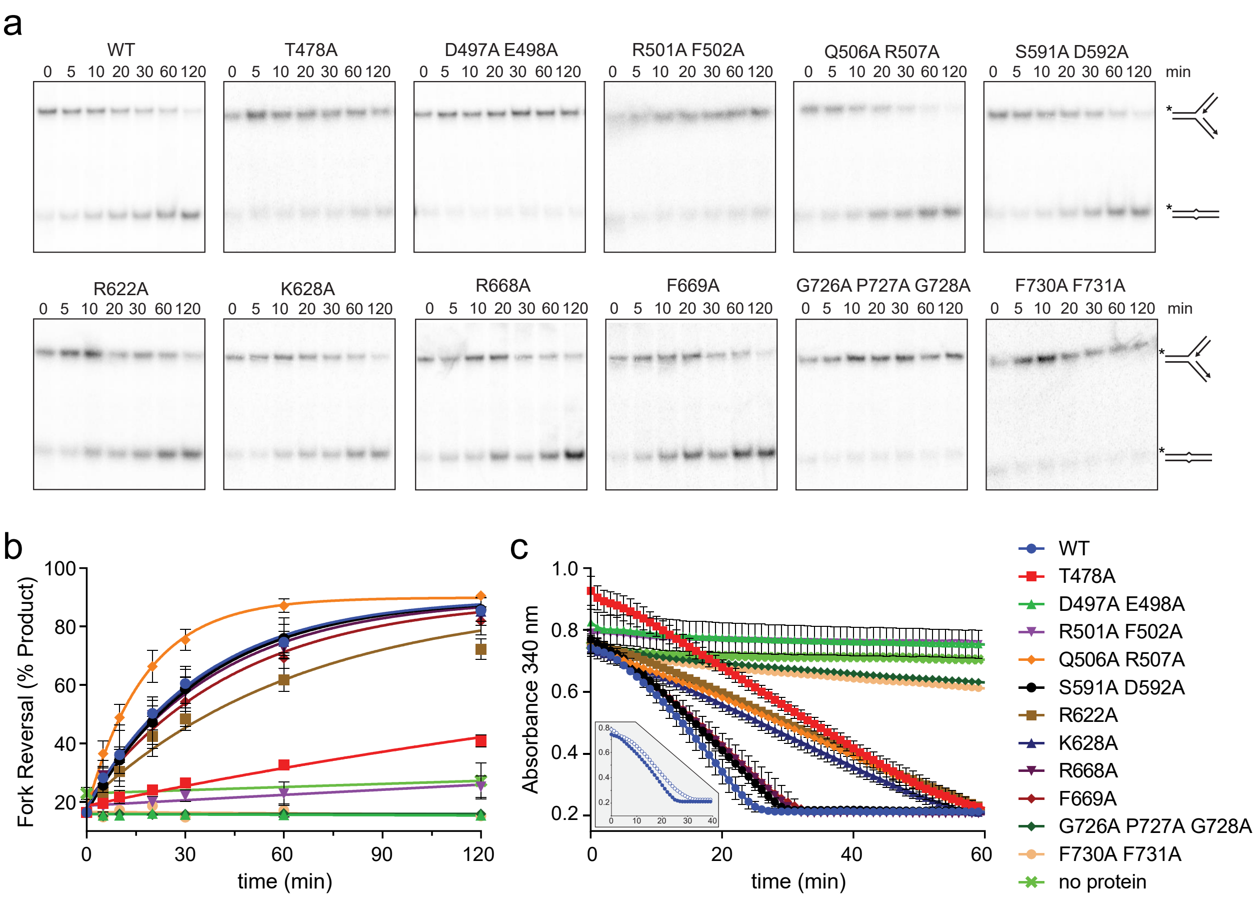

d	ATP hydrolysis (min^{-1})	Fork reversal (×10^{-2} min^{-1})
WT	242 ± 21	2.9 ± 0.1
T478A	135 ± 11	0.3 ± 0.02
D497A E498A	10 ± 2	n.d.
R501A F502A	7 ± 2	0.09 ± 0.02
Q506A R507A	91 ± 5	5.6 ± 0.3
S591A D592A	206 ± 11	2.8 ± 0.2
R622A	87 ± 4	1.5 ± 0.1
K628A	100 ± 4	2.8 ± 0.2
R668A	181 ± 10	2.6 ± 0.1
F669A	183 ± 20	2.2 ± 0.2
G726A P727A G728A	20 ± 4	n.d.
F730A F731A	23 ± 2	n.d.
no protein	8 ± 6	0.06 ± 0.05

Figure S3. Fork reversal and ATP hydrolysis data from TmRecG-His$_6$ mutants. Shown is the raw data used to generate the relative rates in Figure 4b. (a) Representative native PAGE fork reversal data. Lanes are time points in minutes. (b) Quantitation of fork reversal data from three independent experiments (average ± S.D.). (c) ATPase activity from an NADH-coupled assay. The inset shows ATPase activity for WT protein incubated with the immobile Holliday junction used in all ATPase experiments (solid circles) and a reversable fork used in fork reversal assays (open circles). The corresponding rates are 240 min^{-1} (HJ) and 222 min^{-1} (fork). (d) Rates extracted from data shown in panels b and c.

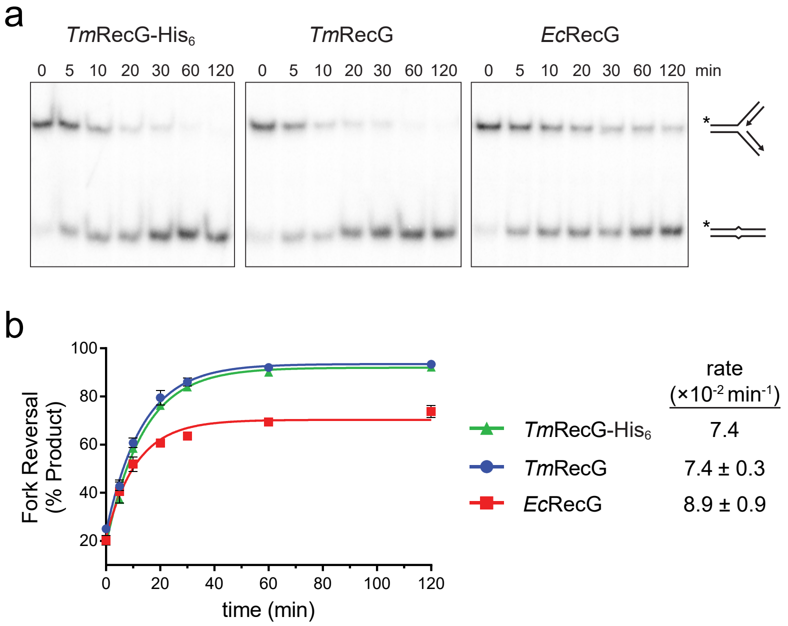

Figure S4. The C-terminal His-tag does not affect RecG activity. (a) Representative native PAGE fork reversal data for a *T. maritima* RecG variant with or without a 3C protease-cleavable C-terminal His_6 tag, compared to wild-type *E. coli* RecG. Lanes are time points in minutes. (b) Quantitation of fork reversal data from three independent experiments (average ± S.D.). The*Tm*-RecG-His_6 experiment was performed once. Rates extracted from fits to the data are shown to the right of the legend.
